# More Doctors Wanted—A Suggestion

**Published:** 1915-05-01

**Authors:** 


*To the Editor of* The Hospital.

### More Doctors Wanted—a Suggestion.

*To the Editor of* The Hospital.

Sir,—Sir Alfred Keogh, the Director-General of Army Medical Service, has issued an appeal for more doctors, but, unfortunately, more doctors are not to be found in any number. It is important at this time of need, therefore, to look carefully into the organisation of our military hospitals with a view to the services of experienced doctors being used to the fullest advantage, and the question at once arises as to whether, at any rate, some of the details at present performed by medical administrators could not be undertaken by experienced non-medical men. The *Times* pointed out recently that there was reason to think that the organisation of medical work was rather wasteful, that cases are recorded in which doctors with one kind of qualification are performing work of another kind—a pathologist giving anæsthetics, etc.

I venture another suggestion: that if medical administrators could be freed from some of the routine clerical work which their office involves, their medical experience would be of untold value in other directions. This suggestion aims simply at transferring numerous details which do not need to be undertaken by a medical man to no less efficient non-medical men. I have reason to think, for instance, that some of the large military hospitals may not even possess a typewriter; certainly from my own observation a great deal of correspondence is personally undertaken by medical administrators, and it would be a saving of valuable time if in the office of each commandant and registrar a clerk and typist were employed to deal with correspondence and other routine matters.

The necessarily hurried mobilisation of some of the new hospitals at the beginning of the campaign undoubtedly accounts for what I think is a misdirection of energy, but in saying this full tribute must be paid to the zeal in which major difficulties have been surmounted. But in the present need for the services of medical men would efficiency not be increased by something being done to employ laymen for clerical work?—Yours faithfully,

"Hospital Officer."

